# Ultra‐Sensitive SPEAR Immunoassay with Cross‐Platform qPCR Consistency for Neurodegenerative Biomarker Measurement

**DOI:** 10.1002/alz70856_106773

**Published:** 2026-01-08

**Authors:** Darren Yang, Xue Jiang, Yunfei Niu, Zixuan Qu, WonHee Kim, Corinne Thomas, Wenjing Jiang, Jeremy Tan, Feng Xuan

**Affiliations:** ^1^ Spear Bio, Woburn, MA, USA; ^2^ SPEAR BIO INC, Woburn, MA, USA

## Abstract

**Background:**

Neurodegenerative diseases such as Alzheimer's disease (AD) require highly sensitive and specific diagnostic tools for early detection and monitoring. Immunoassay‐based detection of disease‐associated biomarkers in biofluids shows promise but faces challenges in sensitivity, reproducibility, and scalability. Our study presents a semi‐automated immunoassay workflow powered by SPEAR (Successive Proximity Extension Amplification Reaction) technology to enhance detection capabilities and enable high‐throughput biomarker analysis. Robustness was demonstrated through consistent results across different qPCR instruments, highlighting the method's adaptability in diverse laboratory settings.

**Method:**

The core SPEAR technology employs a 2‐factor authentication mechanism to minimize background from non‐specific interactions and uses a homogeneous, wash‐free workflow with a real‐time PCR readout. This design facilitates straightforward integration into automation systems without requiring proprietary equipment. The majority of the workflow was automated on a Formulatrix F.A.S.T™ liquid handler using only 1 µL of diluted sample and paired with multiple qPCR instruments to demonstrate cross‐platform consistency. Clinical plasma samples were tested for key AD biomarkers, including phosphorylated tau (*p*‐Tau 217, *p*‐Tau 231), neurofilament light chain (NfL), and glial fibrillary acidic protein (GFAP).

**Result:**

The SPEAR‐based semi‐automated workflow enables high‐precision quantification of low‐abundance biomarkers in single‐digit femtomolar (fM) range. Intra‐ and inter‐assay coefficients of variation (CV) were below 10%, and sample readings were consistent across different qPCR platforms, confirming the assay's robustness and scalability.

**Conclusion:**

This SPEAR‐powered semi‐automated immunoassay workflow advances biomarker quantification for neurodegenerative disease diagnostics by improving sensitivity, reducing operator variability, and supporting high‐throughput processing. Its cross‐platform consistency underscores the assay's versatility in diverse laboratory environments. The platform's ability to detect low‐abundance biomarkers from minimal sample volumes positions it as a valuable tool for both clinical and research applications.

